# Low rank and sparsity constrained method for identifying overlapping functional brain networks

**DOI:** 10.1371/journal.pone.0208068

**Published:** 2018-11-28

**Authors:** Priya Aggarwal, Anubha Gupta

**Affiliations:** Signal Processing and Bio-medical Imaging Lab (SBILab), Indraprastha Institute of Information Technology-Delhi (IIIT-D), New Delhi, India; University of Texas at Austin, UNITED STATES

## Abstract

Analysis of functional magnetic resonance imaging (fMRI) data has revealed that brain regions can be grouped into functional brain networks (fBNs) or communities. A community in fMRI analysis signifies a group of brain regions coupled functionally with one another. In neuroimaging, functional connectivity (FC) measure can be utilized to quantify such functionally connected regions for disease diagnosis and hence, signifies the need of devising novel FC estimation methods. In this paper, we propose a novel method of learning FC by constraining its rank and the sum of non-zero coefficients. The underlying idea is that fBNs are sparse and can be embedded in a relatively lower dimension space. In addition, we propose to extract overlapping networks. In many instances, communities are characterized as combinations of disjoint brain regions, although recent studies indicate that brain regions may participate in more than one community. In this paper, large-scale overlapping fBNs are identified on resting state fMRI data by employing non-negative matrix factorization. Our findings support the existence of overlapping brain networks.

## Introduction

Functional Connectivity (FC) is a widely used measure to quantify relationship between pairs of brain regions [1, 2]. It is utilized to identify functional brain networks (fBNs) and is useful for understanding intrinsic functional organization of human brain [3, 4]. Therefore, accurate construction of FC is one of the most essential tasks to understand the functioning of complex human brain.

Various analytical methods have been proposed for FC modeling from fMRI data such as Pearson correlation (CORR) [1, 5] and the partial correlation (PCORR) [6, 7]. Pearson correlation (CORR) is the most-widely used method for characterizing FC between different brain regions [1, 5]. Despite its popularity, this method is limited because it reveals the pair-wise relationship between two regions without accounting for the influences of other brain regions [8]. Similarly, PCORR method, i.e., the maximum likelihood estimation (MLE) of the inverse covariance matrix, only captures the pairwise information and therefore, does not fully reflect the interactions among multiple brain regions. To solve this problem, recent methods of estimating FC rely on linear relationships between all brain regions and delineate functional relationships by representing one region’s representative time-series as a linear combination of other regions’ time-series. These methods generally impose sparsity on FC coefficients because a brain region may directly interact with only a fewer other brain regions and thus, force insignificant connections to zero using the sparsity constraint [8–10]. However, this solution may yield too sparse fBNs owing to division of actual fBNs into smaller communities and hence, does not guarantee extraction of accurate communities or fBNs. Thus, sparsity constraint alone may not capture the modular structure of brain regions belonging to the same community. To address this problem, some previous studies employed both sparse and dense constraints to characterize FC [8, 9]. It is observed that sparsity constraint helps in extracting sparse inter-network connections and denseness helps in extracting dense intra-network connections.

Apart from considering sparsity and denseness constraints, an informative FC graph is essentially low rank [11–13], which implies that columns and rows of FC are lying in the lower dimensional space, where rows and columns signify brain regions. Low rank constraint provides a modular structure to fBNs, which is closer to the actual real fBNs [3, 13]. However, none of the existing methods of estimating FC utilize low-rank assumption in the formulation. Inspired by this insight, we propose a new multivariate method for learning FC matrix with an assumption that this matrix is reasonably low rank. In addition, we also impose sparsity constraint to learn sparse fBNs as has been explored in the previous studies [8–10].

The proposed method is formulated as a convex minimization problem with *l*^1^-norm (via sparsity convex surrogate) and nuclear norm (via low rank convex surrogate) constraints. We name the proposed method as Low-Rank Multivariate Vector Regression-based Connectivity (LR-MVRC). We utilize popular Alternating Direction Method of Multipliers (ADMM) for solving the newly proposed objective function because it is widely used for solving joint *l*^1^ and nuclear norm problems in different functional magnetic resonance imaging (fMRI) experiments [14, 15].

Second contribution of the proposed work is the identification of overlapping communities. In specific, communities are a group of tightly interconnected brain regions forming functional brain networks. *Overlapping* communities imply that one brain region might be involved in multiple communities. This sounds plausible because one stimulus, say auditory, may stimulate memory and other functional networks apart from the auditory network. This indicates a need for identifying overlapping communities compared to the commonly identified disjoint communities [16, 17]. A few recent studies in fMRI have indicated the existence of overlapping brain communities, for example, popular Independent Component Analysis (ICA) method in fMRI results in overlapping set of clusters [18]. In addition, a few recent studies based on functional connectivity indeed indicate overlap among various functional brain networks and suggest that brain regions may belong to several communities simultaneously [16, 17, 19, 20]. Although several efforts have been made in this direction, the overlapping community structure of brain networks are still largely unclear. Most of these studies are based on matrix factorization approaches to represent FC matrix into lower dimensional space of size *K* (i.e. number of communities), where each one of the *K* dimensions of this space represents association of various brain regions to the corresponding community. Existing methods largely impose external sparsity constraint on this lower dimension space so as to make community inference easy from the perspective of fBNs. However, it is still unclear whether this type of network inference is actually sparse or dense.

Another issue of the existing methods is the utilization of CORR method to build FC, followed by, thresholding on CORR values to retain only a few top connections of FC matrix. This may not be a reasonable assumption. In this work, we utilize full FC matrix to detect overlapping community structure without using any predefined threshold value. In addition, we utilize conventional matrix factorization approach to obtain overlapping fBNs.

Owing to non-negative FC matrix obtained with LR-MVRC, we utilize non-negative matrix factorization (NMF) approach without imposing any external sparsity constraint. NMF is a popular unsupervised machine learning matrix factorization approach [21]. It has been widely used to obtain overlapping communities, especially, in the social networks [22–24]. This method has also been applied to neuroimaging data to localize co-varying structural brain regions [25], to characterize brain tumor heterogeneity [26], and to reveal altered default mode network in Attention Deficit Hyperactivity Disorder (ADHD) [27]. In this paper, NMF is used to obtain overlapping communities or fBNs using fMRI data. The non-negativity property retained in NMF is crucial since the interpretation of results becomes straightforward. We utilize publicly available 1000 Functional Connectomes Project resting state fMRI data to show the effectiveness of the proposed method in extracting fBNs on a group of subjects. Please note that fBNs and communities words are used interchangeably from now onwards.

Salient contributions of the proposed work are as follows:

1. We propose a novel multivariate method for computing FC with sparsity and low-rank constraints.
2. We extract overlapping communities in contrast to non-overlapping communities extracted in general.

To validate the proposed work, it is compared with two state-of-the-art network-based community detection methods: modularity optimization and independent component analysis (ICA). This paper is organized as follows. Data description, proposed LR-MVRC method, its implementation and framework for detecting overlapping communities are presented in Section 2. Section 3 presents results. In the end, discussion and conclusions are presented in Sections 4 and 5, respectively.

## Materials and methods

### Data description and preprocessing

Beijing_Zang resting state fMRI data from 1000 Functional Connectomes Project (http://fcon_1000.projects.nitrc.org/) is utilized in this paper. This data was collected by Beijing Normal University and consists of normal subjects (age range: 18-26 years old) scanned for a duration of 7.5 minutes resulting in 225 brain volumes. Each brain volume consists of an acquisition of 33 axial brain slices with dimension 64 × 64. This data has Echo Time (TE) equal to 30 ms and Repetition Time (TR) equal to 2000 ms. We considered all 198 subjects of this dataset in this manuscript.

We carried out standard preprocessing using SPM12 (http://www.fil.ion.ucl.ac.uk/spm/software/spm12/) toolbox as specified in [8]. Preprocessing starts with the removal of first 10 volumes. This initial step is necessary to allow the magnetization to reach the steady state. After this step, other remaining brain volumes are slice time corrected using the middle slice as a reference followed by motion correction. Motion correction ensures reduction of head motion artifact from the signal. Further, spatial normalization onto the Montreal Neurological Institute (MNI) space is performed to facilitate group-level comparisons, followed by, smoothing with a Gaussian kernel with 4 mm full width half maximum (FWHM). Finally, we regressed out nuisance variables (6 head motion parameters, average cerebrospinal fluid (CSF) signal from ventricular masks, and average white matter signal from white matter mask) from each voxel’s time series and performed bandpass filtering in the frequency range of 0.01 to 0.1 Hz to reduce low frequency drift and high frequency noise.

After preprocessing, the whole brain data is parcellated into 90 anatomical regions of interests (ROIs) via automated anatomical labeling (AAL) atlas [28]. In order to find the region-representative time series for every ROI, we averaged time-series of all voxels belonging to the same ROI. This resulted into a matrix **X** of dimension *T* × 90, where *T* denotes the number of time points (or the number of brain volumes) such as 215 for the given fMRI data. Next, we normalized each column of **X** to obtain unit normalized time-series.

### Proposed LR-MVRC

Consider a matrix **X** of dimension *T* × *N*, where *T* denotes the number of time points and *N* denotes the number of region of interests (ROIs). Each column of **X** signifies unit normalized time-series of one brain region. Given this matrix, we require to compute the functional connectivity matrix of dimension *N* × *N*. Recently proposed Multivariate Vector Regression-based Connectivity (MVRC) method of identifying FC regresses time series of all regions (i.e. columns of **X**) onto the time series of other regions multiplied by an FC matrix as $X=X\tilde{W}$ [8]. In addition, this method employs elastic-net penalty onto the FC matrix comprising of both *l*^1^ and *l*^2^ norms. However, to incorporate modular structure of fBNs into FC formulation, we consider nuclear norm constraint along with *l*^1^ norm constraint in the computation of this matrix and name the propose method as Low-Rank Multivariate Vector Regression-based Connectivity (LR-MVRC). We formulate the proposed LR-MVRC objective function as:

$$\underset{\tilde{\text{W}}}{min}\;\frac{1}{2}{∥\text{X}-\text{X}\tilde{W}∥}_{F}^{2}+\mu_{1}{∥\tilde{\text{W}}∥}_{1}+\mu_{2}{∥\tilde{\text{W}}∥}_{*},\;\text{s}.\text{t}.\;\;diag(\tilde{\text{W}})=0,$$ (1)

where *μ*_1_ and *μ*_2_ are the regularization parameters associated with *l*^1^ and nuclear norms terms, respectively. Nuclear norm minimization implies *l*^1^ penalty on singular values of matrix $\tilde{W}$ that supports this matrix to be low-rank. $diag(\tilde{W})=0$ term ensures no self connections in the matrix $\tilde{W}$. Finally, symmetric adjacency matrix from $\tilde{W}$ is computed as $A=(\tilde{\left|W\right|}+{\tilde{\left|W\right|}}^{T})/2$.

### Implementation of LR-MVRC

Next, we introduce the algorithm to solve LR-MVRC problem. We utilize ADMM [29] that splits Eq (1) into multiple subproblems that are easier to solve. We introduce two auxiliary variables **Z**_1_ and **Z**_2_ for the *l*^1^-norm and nuclear norm terms in Eq (1) as:

$$\begin{array}{c} \begin{array}{c} \underset{\tilde{W}}{min}\;\frac{1}{2}{∥X-X\tilde{W}∥}_{F}^{2}+\mu_{1}∥Z_{1}{∥}_{1}+\mu_{2}∥Z_{2}{∥}_{*}\\ \;\text{s}.\text{t}.\;\;Z_{1}=\tilde{W},Z_{2}=\tilde{W},diag\left(\tilde{W}\right)=0. \end{array} \end{array}$$ (2)

The augmented Lagrange function for Eq (2) can be written as:

$$\begin{array}{c} \begin{array}{c} L\left(\tilde{W},Z_{1},Z_{2},Y_{1},Y_{2}\right)=\frac{1}{2}{∥X-X\tilde{W}∥}_{F}^{2}+\mu_{1}∥Z_{1}{∥}_{1}\\ +\mu_{2}∥Z_{2}{∥}_{*}+\frac{\beta_{1}}{2}{∥Z_{1}-\tilde{W}+\frac{Y_{1}}{\beta_{1}}∥}_{F}^{2}+\frac{\beta_{2}}{2}{∥Z_{2}-\tilde{W}+\frac{Y_{2}}{\beta_{2}}∥}_{F}^{2}, \end{array} \end{array}$$ (3)

where *β*_1_, *β*_2_ > 0 are the penalty parameters and **Y**_1_, **Y**_2_ are the Lagrangian multiplier matrices. The above equation consists of three variables $\tilde{W}$, **Z**_1_ and **Z**_2_. This is to note that during the iterative learning for the solution of $\tilde{W}$ in Eq (3), $diag(\tilde{W})$ is kept to zero.

ADMM splits Eq (3) into three subproblems as described below. Each subproblem may be treated as minimization over one variable while fixing the other two variables.

**Subproblem 1**: Solving $\tilde{W}$:

$$\underset{\tilde{W}}{min}\;\frac{1}{2}{∥X-X\tilde{W}∥}_{F}^{2}+\frac{\beta_{1}}{2}{∥Z_{1}-\tilde{W}+\frac{Y_{1}}{\beta_{1}}∥}_{F}^{2}+\frac{\beta_{2}}{2}{∥Z_{2}-\tilde{W}+\frac{Y_{2}}{\beta_{2}}∥}_{F}^{2}.$$ (4)

Update of $\tilde{W}$, while other variables are fixed, is performed by solving the above equation as below:

$$\tilde{W}={\left(X^{T}X+\left(\beta_{1}+\beta_{2}\right)I\right)}^{-1}\left(X^{T}X+\beta_{1}Z_{1}+Y_{1}+\beta_{2}Z_{2}+Y_{2}\right),$$ (5)

where **I** is an identity matrix. The diagonal elements of $\tilde{W}$ obtained from Eq (4) are replaced with zeros.

**Subproblem 2**: Solving **Z**_1_:

$$\underset{Z_{1}}{min}\;\mu_{1}∥Z_{1}{∥}_{1}+\frac{\beta_{1}}{2}{∥Z_{1}-\tilde{W}+\frac{Y_{1}}{\beta_{1}}∥}_{F}^{2}.$$ (6)

Update of **Z**_1_, while other variables are fixed, can be done using soft thresholding as:

$$Z_{1}=Soft_{2\mu_{1}/\beta_{1}}\left(\tilde{W}-\frac{Y_{1}}{\beta_{1}}\right),$$ (7)

where *Soft* is the shrinkage thresholding operator defined as [30]:

$$Soft_{\alpha }\left(\nu \right)=sgn\left(\nu \right)\text{max}\left(0,|\nu |-\alpha \right),$$ (8)

where *sgn* denotes the signum value and *max* denotes the maximum value.

**Subproblem 3**: Solving **Z**_**2**_:

$$\underset{Z_{2}}{min}\;\mu_{2}∥Z_{2}{∥}_{*}+\frac{\beta_{2}}{2}{∥Z_{2}-\tilde{W}+\frac{Y_{2}}{\beta_{2}}∥}_{F}^{2}.$$ (9)

Global minimum of convex nuclear norm minimization is obtained by soft thresholding on singular values, known as singular value thresholding (SVT) [31]. Hence, update of **Z**_2_ while other variables are fixed, can be carried out using soft thresholding on singular values of **Z**_2_ as:

$$Z_{2}=\text{SV}{\text{T}}_{2\mu_{2}/\beta_{2}}\left(\tilde{W}-\frac{Y_{2}}{\beta_{2}}\right),$$ (10)

where SVT is defined as:

$$\text{SV}{\text{T}}_{\alpha }\left(\nu \right)=U\times diag\left(SOFT_{\alpha }\left(\nu \right)\right)\times V^{T},$$ (11)

and singular value decomposition of **Z**_2_ is defined as **U** × *diag*(*ν*) × **V**^*T*^.

The iterations of LR-MVRC, with update of variables, is described in *Algorithm 1*.

**Algorithm 1**. LR-MVRC problem

**Input**: Data matrix **X** and parameters *μ*_1_, *μ*_2_.

**Initialize**: *β*_1_ = *β*_2_ = 0.1, *β*_*max*_ = 10^10^, *ρ* = 1.1, $Y_{1}=Y_{2}=\tilde{W}=Z_{1}=Z_{2}=0$.

**while** convergence criteria not met **do**

 1: Fix the other variables and update $\tilde{W}$ by Eq (4)

 2: Fix the other variables and update **Z**_1_ by Eq (6)

 3: Fix the other variables and update **Z**_2_ by Eq (9)

 4: Update the multipliers by

$$Y_{1}=Y_{1}+\beta_{1}\left(Z_{1}-\tilde{W}\right),Y_{2}=Y_{2}+\beta_{2}\left(Z_{2}-\tilde{W}\right)$$

 5: Update *β*_1_ = *min*(*β*_*max*_, *ρβ*_1_) and *β*_2_ = *min*(*β*_*max*_, *ρβ*_2_).

**end while**

**Output**: $A=(\tilde{\left|W\right|}+{\tilde{\left|W\right|}}^{T})/2$.

### Functional brain network identification

After computing the adjacency matrix, next task is to identify communities or fBNs. Various methods have been proposed to detect communities from FC matrix [32]. Among them, modularity is one of the most popular methods [33]. The widely used modularity method implemented in Brain Connectivity Toolbox [33] yields disjoint communities, i.e., one brain region is part of only one community, although recent studies in fMRI [16] demonstrate that one brain region may participate in multiple communities. Thus, we identify overlapping communities of ROIs. In the next section, we describe the method for detecting overlapping communities.

#### Detection of overlapping communities

In this work, we utilize NMF technique to obtain overlapping communities. NMF is a feature extraction and dimensionality reduction method of machine learning, which has been adapted to community detection recently [22–24]. This technique factorizes a non-negative input FC matrix **A**, approximately, into a product of two non-negative matrices as:

A≃PQ. (12)

The above factorization is carried out with a particular rank *K* so that **P** is of dimension *N* × *K* and **Q** is of dimension *K* × *N*. This factorization can be viewed as a representation of data in a lower (*K*) dimensional space. In NMF, matrices **P** and **Q** are updated iteratively to improve the approximation to **A**, while maintaining non-negative matrix entries throughout. For a given *K*, the algorithm runs iteratively until it finds a good approximate factorization or the stop criterion is met. For a symmetric data matrix **A**, the factors **P** and **Q** can be considered as **Q** = **P**^*T*^.

In particular, NMF algorithm minimizes the cost function, representing the approximation error between the actual data **A** and the reduced dimension reconstruction of the data, i.e., **PQ**. One of the popular cost function is the squared Euclidean distance as described below:

$$L\left(P,Q\right)=\frac{1}{2}\sum_{i}\sum_{j}{\left(A_{ij}-{\left[\text{PQ}\right]}_{ij}\right)}^{2}.$$ (13)

The minimization of the above cost function w.r.t. **P** ≥ 0 and **Q** ≥ 0 is a nonconvex problem. This approach can be interpreted as a maximum likelihood estimation with additional non-negativity constraints. However, the implementation details which are necessary to ensure decreasing cost function under the non-negativity constraints seem to be rather complex [34]. In addition, without any other constraint or prior to this problem, solution may lead to unstable convergence, and therefore, good initial values are necessary for more sophisticated NMF algorithms [35, 36]. Different iterative algorithms have been proposed to solve this problem [35]. In general, at each iteration of these algorithms, one matrix is considered to be fixed and the other one is estimated. This process continues until the convergence is achieved. The main idea behind these approaches is that by fixing one matrix, the estimation of the other matrix becomes a convex problem that can be solved easily.

Since NMF, in general, has no unique solution, it is necessary to introduce some additional constraints reflecting some prior knowledge. To this end, recently a Bayesian approach of NMF is proposed that imposes prior distributions on the matrices and leads to unique convergence to solution [37]. In recent overlapping communities detection work [22], it is assumed that the pairwise interaction *A*_*ij*_ is generated by a Poisson distribution with rate ${\hat{A}}_{ij}=\sum_{k}P_{ik}Q_{kj}$ and shrinkage prior on *P*_*ik*_ and *Q*_*kj*_ with hyper-parameter *β*_*k*_. These additional constraints help to induce some kind of uniqueness to the NMF solution. Further, this Bayesian NMF approach maximizes posterior density under non-negative constraints. Keeping in view the success of this Bayesian NMF approach for overlapping communities detection, we have utilized algorithm presented in [22] to solve for NMF in this work. This algorithm starts with random initial matrices **P** and **Q**, with non-negative weights chosen from a uniform random distribution on the interval [0, 1]. In brief, these matrices are updated iteratively as

$$\begin{array}{c} \begin{array}{c} Q\leftarrow \left(\frac{Q}{P^{T}1+BQ}\right).\;\left[P^{T}\left(\frac{A}{PQ}\right)\right],\\ P\leftarrow \left(\frac{P}{1Q^{T}+PB}\right)\;.\;\left[\left(\frac{A}{PQ}\right)Q^{T}\right], \end{array} \end{array}$$ (14)

where $B\in R^{K\times K}$ is a matrix with hyper-parameters *β*_*k*_ on the diagonal. For more details of this algorithm, one may refer to [22]. Code of this algorithm is available online at https://github.com/ipsorakis/commDetNMF. We have set the number of maximum iterations to 100 as has been done in [22]. This is to note that we ran the algorithm ten times with random initial matrices. The solution converged to the same output matrices with some columns exchanged.

Most experiments show that the matrix **P** represents the final clustering partition. In our work, we mainly utilized matrix **P** to determine overlapping communities of the input FC matrix. This is to note that each column of matrix **P** defines one community and hence, *K* denotes the total number of communities. Each column of **P** indicates the extent to which any brain region *i* belongs to that community. Thus, we compared each column of **P** with a threshold and if the value of region *i* is more than the threshold, region *i* is considered to be a part of that community. Similarly, if a region’s value is less than the threshold, we delete that region from the corresponding community. Further details about selection of this threshold is provided in the Results Section.

Thresholding of each column of **P** independently allows brain regions to be a part of multiple communities simultaneously and hence, detects overlapping communities. The NMF approach of detecting communities is already used in the literature of social networks. However, their use in brain networks is limited so far.

#### Choosing number of *K* communities

NMF algorithm requires a given value of dimensionality *K* that is an important input used in matrix factorization. However, determining the value of *K* is a challenge in most community discovery algorithms because the number of communities *K* is not known in advance. If *K* is too small, some communities will be very large (in terms of more number of regions) with random grouping of ROIs. On the contrary, if *K* is too large, communities will be randomly scattered and will be very small.

To determine the number of communities *K*, we used NMF for two different numbers of communities 8 and 15. We chose 8 number of communities because, in general, the following eight brain networks are observed in the resting state fMRI data: Visual Network (VN), Somato-motor Network (SMN), Auditory Network (AN), Cognitive Control Network (CCN), Bilateral Limbic Network (BLN), Language Network (LN), Subcortical Network (SCN), and Default Mode Network (DMN). We chose to extract 15 number of communities because we would like to ascertain if choosing a number larger than the expected number of communities yields scattered networks.

## Results

We utilized **X** and computed adjacency matrix using Eq (1) for all subjects. We used *μ*_1_ = 0.25 in Eq (1) as was utilized previously in [8] and the value of *μ*_2_ is decided empirically to be equal to 0.1. This value is decided based on the correspondence of identified fBNs with the ground truth atlas labels. We averaged LR-MVRC FC matrices of all subjects and utilized NMF to obtain communities.

### Comparison of FC methods

In this section, we evaluate the feasibility and the robustness of the proposed LR-MVRC method w.r.t. traditional methods discussed below.

- **Pearson correlation (CORR)**: This is simply a pairwise correlation between two brain regions’ time series. Pearson correlation coefficients are computed between all 90 ROIs (extracted from AAL atlas), resulting in a 90 × 90 FC matrix. All negative values are flipped to positive values and all diagonal entries are set to zeros, as is usually done in fMRI FC analysis using CORR [38].
- **Partial correlation (PCORR)**: Partial correlation values are assumed to be equivalent to the inverse of covariance matrix and also known as the precision matrix. Thus, first the covariance matrix is computed. Next, inverse of this matrix provides partial correlation values. However, computation of inverse is itself challenging because of low rank nature of the covariance matrix. Generalized inverse or pseudo-inverse methods have been generally used to compute partial correlation.
- **MVRC method** [8]: MVRC method has been explained in methods section and is implemented as specified in [8].

We averaged each method’s FC matrices across all subjects and compared them w.r.t. various graph theoretical measures [39]. Degree was calculated as the sum of weighted edges connecting to a node *i* as *D*_*i*_ = ∑_*j*_ **A**_*ij*_. Similarly, participation coefficient (PC) arising from modularity community assignment was computed as $P_{i}=1-\sum_{s=1}^{N_{m}}{\left(\frac{D_{is}}{D_{i}}\right)}^{2}$, where *D*_*is*_ is the number of edges of node *i* to nodes in module *s*, *D*_*i*_ is the degree of node *i*, and *N*_*m*_ is the total number of modules in the graph. We also computed another graph theoretical measure, i.e., betweenness centrality (BC), averaged over all 90 AAL brain regions, whose high value signifies the network to be highly central or modular. In fact, it is a network centrality measure that represents the fraction of all shortest paths in the network that pass through a given node [33].

LR-MVRC matrix in this study is extracted using *l*^1^ and low rank constraints, while MVRC FC matrix in [8] was extracted using *l*^1^ and *l*^2^ constraints. As a result, we noticed that while MVRC had more connections due to the denseness imposed by *l*^2^ norm constraint, LR-MVRC had fewer higher magnitude connections compared to MVRC due to the low rank constraint. However, these fewer connections of LR-MVRC weighed higher than those of MVRC. In addition, we observed same degree in both the matrices, LR-MVRC FC and MVRC FC, since we did not apply any thresholding on FC matrices. Participation coefficients (of size 90 that is equal to the number of AAL brain regions) arising from the modularity community assignment using both the methods are statistically compared using the two-sample *t*-test with significance level of 0.05. Altered PC were observed between both the matrices (*p*<0.05). Further, we observed BC of LR-MVRC to be more (=128) compared to that of MVRC (=96). In conclusion, these findings indicate that the LR-MVRC FC matrix is more modular or central compared to MVRC FC.

Further, on comparing LR-MVRC method with the CORR and PCORR methods, we observed same degree with both the matrices, since we did not apply any thresholding on FC matrices. In addition, we observed PC using CORR and PCORR methods to be statistically different w.r.t. the proposed LR-MVRC method, suggesting that the FC matrices obtained by the three methods are statistically different (*p*<0.05). These results are consistent with the findings on LR-MVRC and MVRC methods as discussed previously, although this is to note that we obtained fewer number of communities using modularity with CORR and PCORR methods with no community signifying any functional brain network structure. Further details regarding this comparison is presented in the next section. Furthermore, analysis of the average BC showed lesser values among the CORR and PCORR methods compared to the proposed LR-MVRC method. These results indicated that there are structural differences among the networks constructed by the three methods.

#### Competing methods

Modularity optimization is a leading method aimed at finding optimal non-overlapping partitions of fBNs based on modularity function [40]. Unlike NMF that finds overlapping communities, modularity optimization results in non-overlapping communities. We performed modularity optimization on the averaged LR-MVRC adjacency matrices by using the Louvain method implemented in the Brain Connectivity Toolbox [33].

Principal and independent component analysis (PCA, ICA) are some other matrix factorization methods commonly used for projecting data into lower dimensional representation, from which the overlapping communities structure could be identified [41]. These factorization methods impose different constraints or priors in order to obtain a solution, e.g., components must be orthogonal in PCA and must be independent in ICA. The ICA method is being extensively used in fMRI and it computes independent components directly on fMRI time-series and identifies overlapping communities. In this paper, fMRI time-series of all subjects are concatenated temporally and the dimensionality is reduced by using PCA [18]. PCA is generally used before ICA for dimensionality reduction. The resulting fMRI time-series data is fed into the fastICA algorithm [42] implemented by the FastICA software (https://research.ics.aalto.fi/ica/fastica/).

To further test the performance of the proposed LR-MVRC FC identification method, all fBNs identification methods, i.e., NMF and modularity optimization are also carried out on the FC matrix constructed using the Pearson correlation (CORR). Specifically, for each pair of ROIs, Pearson correlation is used as a metric to compute the FC. Next, we averaged CORR adjacency matrices of all subjects and utilized it to obtain communities.

#### Results on fMRI dataset

**Fig 1:**
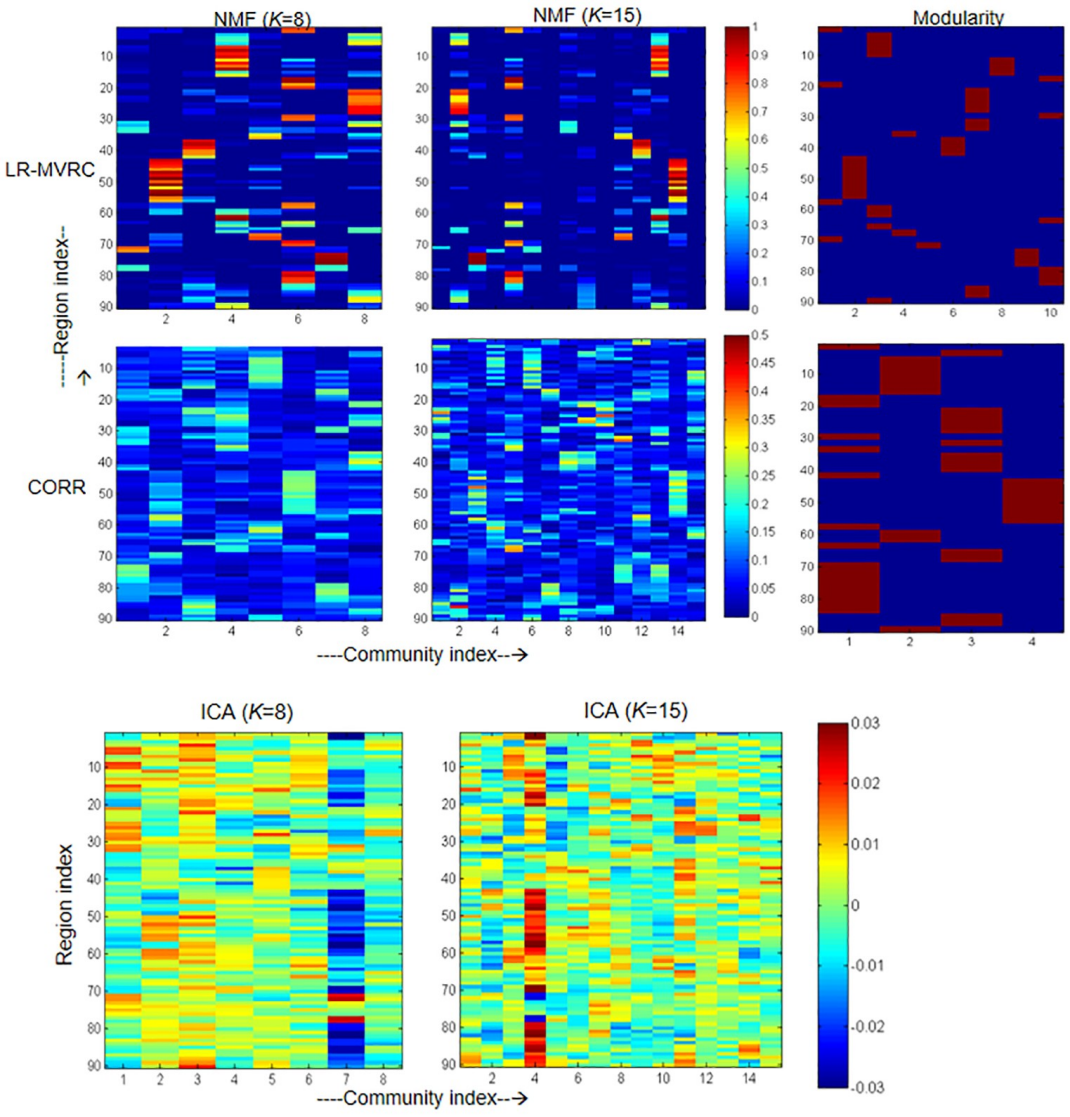
**First row**: **Community structure obtained using LR-MVRC and CORR matrices with NMF and modularity optimization methods**. **Second row**: **Community structure obtained using ICA with *K* = 8 and 15 in the first and second columns, respectively**. In the first row, first two columns represent the matrix **P** obtained from LR-MVRC and CORR with *K* = 8 and 15 in NMF, respectively. The third column represents modularity optimization results on both LR-MVRC and CORR based adjacency matrices.

Based on the analyses of LR-MVRC and CORR FC matrices, the overall community structure obtained by NMF and modularity are shown in Fig 1. The matrices **P** obtained using LR-MVRC and CORR matrices with *K* = 8 and 15 in NMF are shown in first two columns of Fig 1. Visually, LR-MVRC matrices yield sparse overlapping community structures, while the ones derived using CORR matrices are also overlapping but are extremely dense. More sparsity, or in other words, fewer high valued coefficients in each column of matrix **P** results in accurate fBN identification by applying thresholding as described in the previous section. We considered mean plus standard deviation of each column of matrix **P** as a threshold value for that column or community. Modularity optimization method (refer to third column of Fig 1) achieves greatest sparsity among all methods, but this method results in non-overlapping communities. This is to note that this method does not require pre-defined number of communities unlike the NMF method. Using this method, CORR based FC matrix could identify only 4 communities compared to 10 communities detected with LR-MVRC based FC matrix.

In the second row of Fig 1, we show the community structure derived by ICA. The first and the second columns of this row display the overall community structures identified with *K* = 8 and 15, respectively. For ICA method, group-level community structure is derived by concatenating fMRI time-series of all subjects. Here, the number of communities *K* for ICA methods were set according to the values chosen for NMF method in this paper. Visually, the communities derived by ICA show an overlapping structure, but they tend to produce a much denser result with only a few high or negative coefficients values. It reveals that ICA method achieves a moderate similarity to NMF method since this method works directly on the time-series instead of first extracting FC matrix as is done in the NMF method.

**Fig 2:**
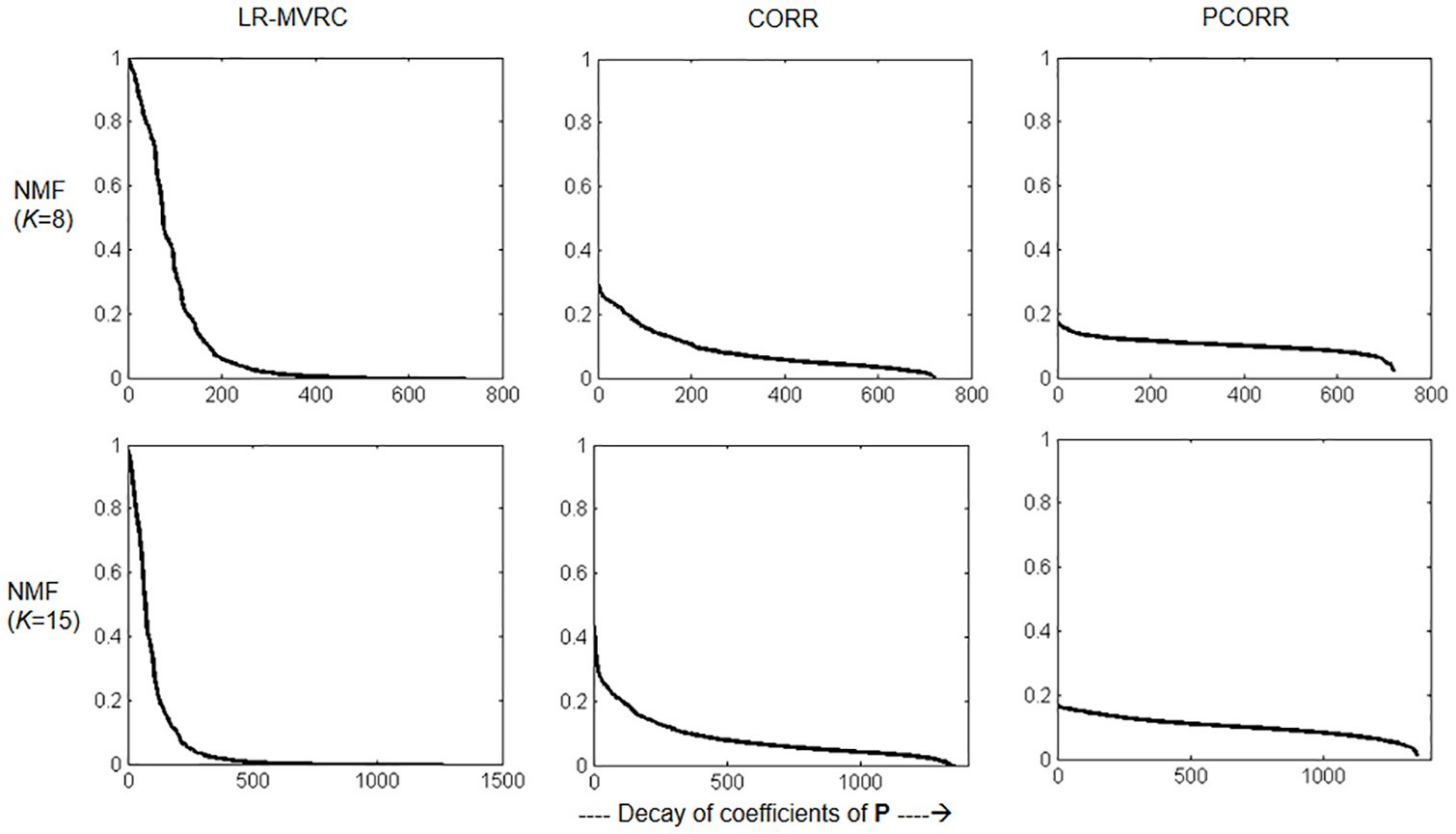
Illustration of sparsity of matrix P obtained with NMF. Plot of sorted coefficients of matrix **P**, obtained using LR-MVRC, CORR and PCORR matrices with different values of *K* in NMF.

To depict sparsity differences between NMF results of LR-MVRC, CORR and PCORR more accurately, we plot sorted coefficients of matrix **P** in Fig 2. We plot results for both 8 and 15 value of *K* in NMF. From this figure, we observe that for each value of *K*, LR-MVRC based results are more sparse with only a few non zero coefficients compared to those extracted from CORR and PCORR FC matrices.

**Fig 3:**
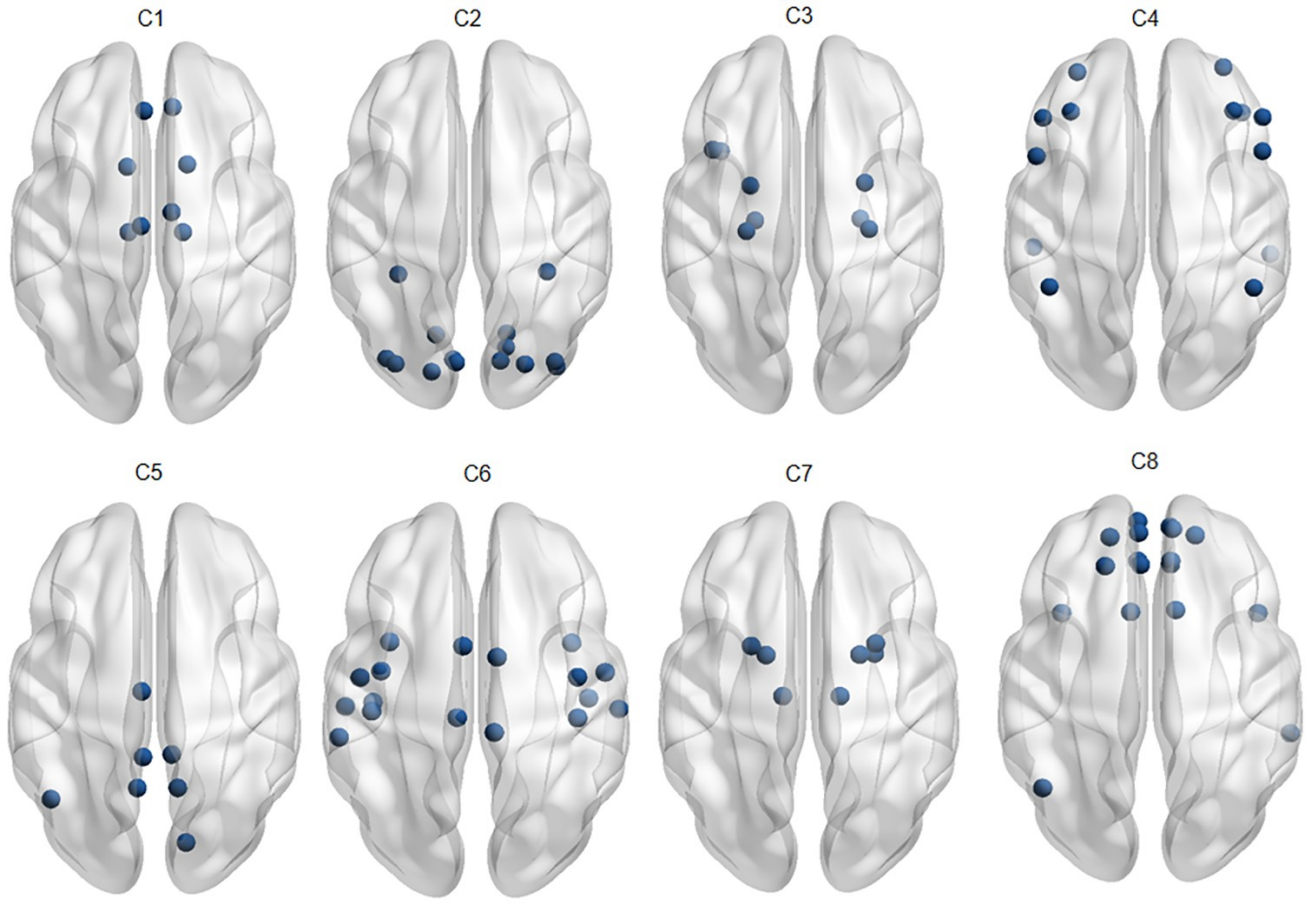
Overlapping communities derived from LR-MVRC. This figure displays the communities derived from the LR-MVRC based adjacency matrix via thresholding of matrix **P** obtained with NMF (*K* = 8). Eight communities derived from LR-MVRC matrix shown in this figure refer to default mode and subcortical (C1), visual (C2), bilateral limbic (C3), cognitive control and default mode (C4), default mode and visual (C5), auditory and motor (C6), subcortical (C7), and default mode and bilateral limbic (C8) networks.

**Fig 4:**
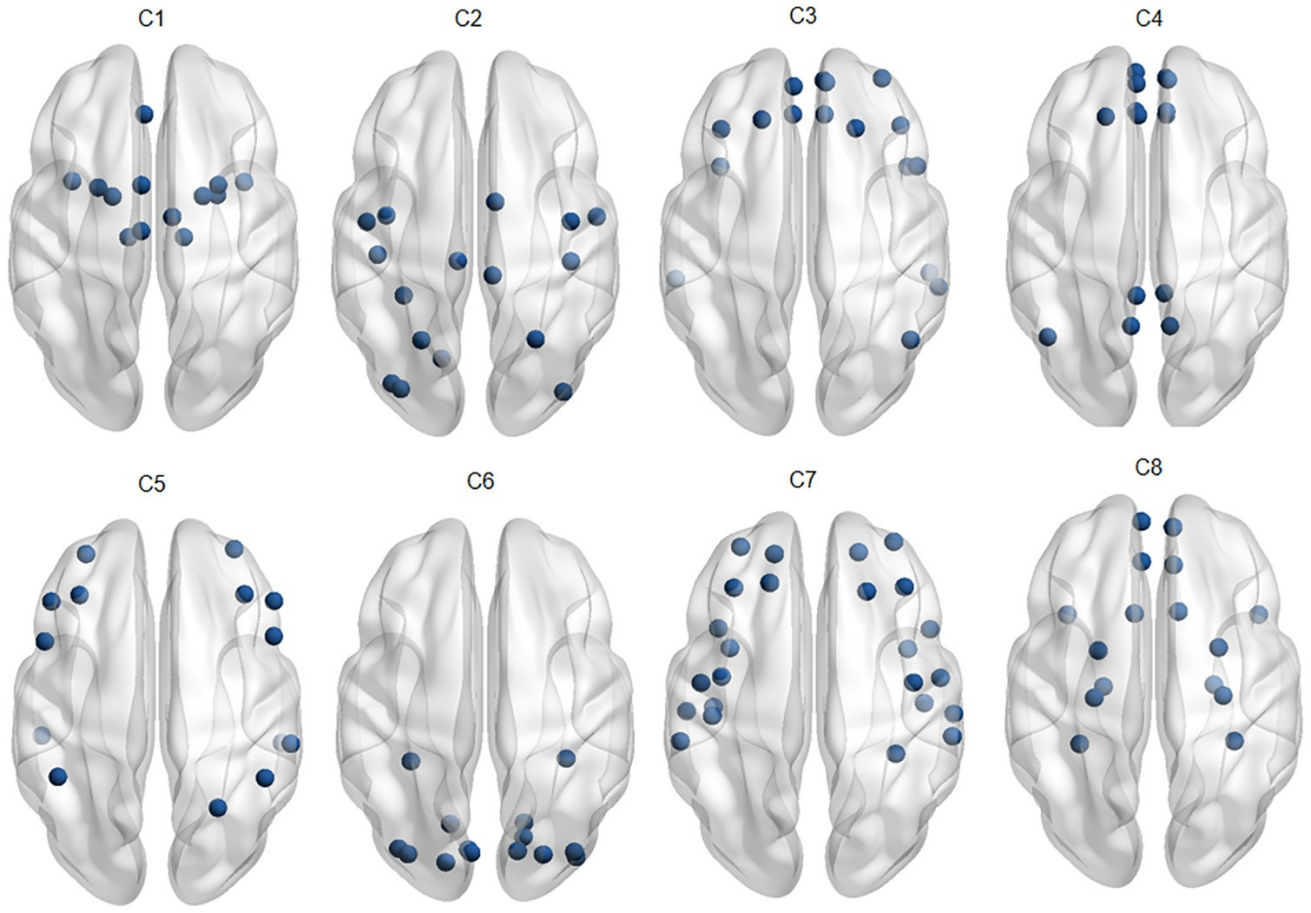
Overlapping communities derived from CORR. This figure displays the communities derived using CORR based matrix via thresholding of matrix **P** obtained with NMF (*K* = 8).

Further, to explore the neuro-physiological interpretations of identified communities with LR-MVRC (refer to Table 1) and CORR, we mapped the identified communities corresponding to *K* = 8 onto the human brain, as shown in Figs 3 and 4. Fig 3 displays the communities derived by LR-MVRC based on the thresholding on matrix **P**. Eight communities derived by LR-MVRC shown in this figure refer to default mode and subcortical (C1), visual (C2), bilateral limbic (C3), cognitive control and default mode (C4), default mode and visual (C5), auditory and motor (C6), subcortical (C7), and default mode and bilateral limbic (C8) networks. These networks are highly consistent with several well-recognized resting state fBNs discovered by previous studies [43–46]. In essence, two communities (C2, C5) are related to the visual information processing, one community (C6) is related to the auditory information processing, four communities (C1, C4, C5 and C8) are associated with the well-known default mode network (DMN), and one (C6) is associated with the motor network. C1 and C7 correspond to the subcortical network. In addition, bilateral limbic network (C3 and C8) and cognitive control network (C4), associated with high-order brain functions are readily identified. Whereas, it is noted from Fig 4 that ROIs associated with different fBNs are clubbed in a single community, showing random grouping of ROIs. This causes difficulty in the interpretation of identified communities as valid fBNs.

**Table 1:** Brain networks identified using LR-MVRC with *K* = 8 in NMF. Fourth column represent AAL atlas ROI indices belonging to the community *K*, whereas second and third columns represent the associated brain networks and number of ROIs in that community, respectively.

Community	Networks	No. of nodes	AAL Regions
1	default mode and subcortical	8	31, 32, 33, 34, 71, 72, 77, 78
2	visual	14	43-56
3	bilateral limbic	8	37-42, 83, 87
4	cognitive control and default mode	13	7-14, 16, 61, 62, 89, 99
5	default mode and visual	7	33, 35, 36, 46, 65, 67, 68
6	motor and auditory	17	1, 2, 17-20, 29, 30, 57, 58, 63, 69, 70, 79-82
7	subcortical	7	42, 73-78
8	default mode and bilateral limbic	17	3, 5, 6, 21-28, 31, 32, 65, 86-88

**Fig 5:**
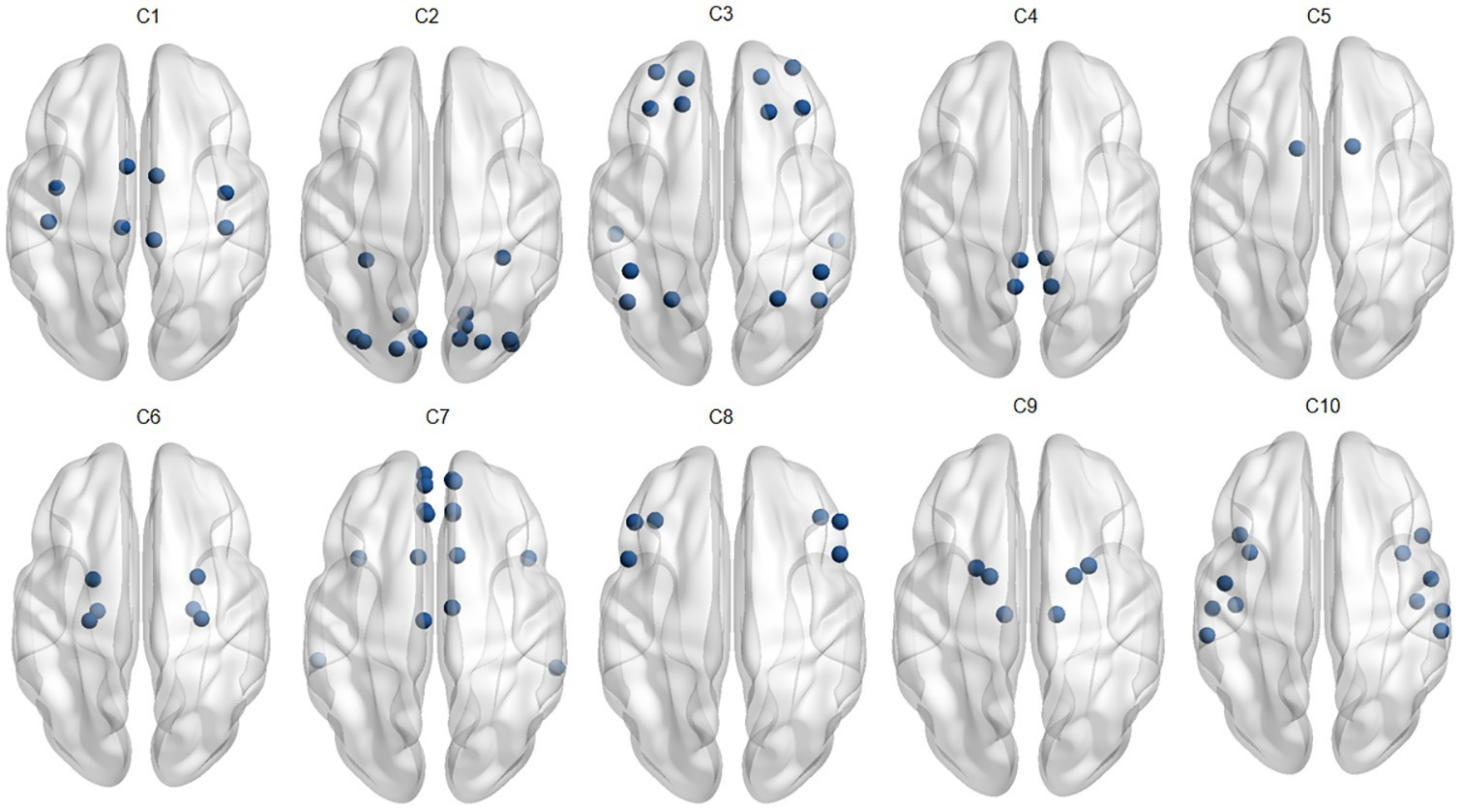
Non overlapping communities derived from LR-MVRC. This figure displays the communities derived using LR-MVRC based matrix via modularity optimization. Ten communities derived by LR-MVRC shown in this figure refer to motor (C1), visual (C2), default mode and cognitive control (C3), default mode (C4), subcortical (C5), bilateral limbic (C6), default mode and bilateral limbic (C7), cognitive control (C8), subcortical (C9) and auditory networks.

Non overlapping communities derived from LR-MVRC (see Fig 5) refer to motor (C1), visual (C2), default mode and cognitive control (C3), default mode (C4), subcortical (C5), bilateral limbic (C6), default mode and bilateral limbic (C7), cognitive control (C8), subcortical (C9) and auditory networks, which are consistent with the overlapping communities detected with LR-MVRC method (see Fig 3).

Apart from considering *K* = 8 in NMF with LR-MVRC, we also present results with *K* = 15 in Table 2. Fifteen communities derived by LR-MVRC shown here refer to default mode and subcortical (C1), default mode and bilateral limbic (C2), bilateral limbic and subcortical (C3), default mode and subcortical (C4), motor and auditory (C5), motor and subcortical (C6), default mode and subcortical (C7), default mode and subcortical (C8), coginitive control, bilateral limbic and default mode (C9), default mode and subcortical (C10), default mode (C11), bilateral limbic (C12), cognitive control and default mode (C13), visual (C14), and default mode and subcortical (C15) networks, which are consistent with several well-recognized fBNs discovered by previous studies. This is to note that the similarity observed in the four communities (C4, C7, C10 and C15) in Table 2 points to the fact that lesser value of *K* should have been used in the method. A value larger than required shows repeated communities, signifying that no better partitioning can happen. This observation can work as an initial criterion to decide the parameter *K* in the NMF method.

**Table 2:** Brain networks identified using LR-MVRC with *K* = 15 in NMF. Fourth column represent AAL atlas ROI indices belonging to the community *K*, whereas second and third columns represent their corresponding associated brain networks and number of ROIs in one community, respectively.

Community	Networks	No. of nodes	AAL Regions
1	default mode and subcortical	3	33, 71, 78
2	default mode and bilateral limbic	15	3, 5, 6, 21-28, 31, 32, 87, 88
3	bilateral limbic and subcortical	7	42, 73-78
4	default mode and subcortical	3	34, 72, 77
5	motor and auditory	17	1, 2, 17-20, 29, 30, 57, 58, 63, 69, 70, 79-82
6	motor and subcortical	10	1, 2, 57, 58, 69-72, 77, 78
7	default mode and subcortical	3	34, 72, 77
8	default mode and subcortical	8	3, 23, 31-34, 77, 78
9	cognitive control, bilateral limbic and default mode	12	15, 16, 56, 66, 83-90
10	default mode and subcortical	3	34, 72, 77
11	default mode	6	35, 36, 46, 65, 67, 68
12	bilateral limbic	9	37-42
13	cognitive control and default mode	12	7-16, 61, 62, 90
14	Visual	14	43-56
15	default mode and subcortical	3	34, 72, 77

We further looked regions belonging to more than one community in LR-MVRC based NMF results. It reveals that regions involved in more than one community span over default mode, subcortical, bilateral limbic and visual networks. By contrast, regions related to sensori-motor and auditory participate in fewer communities.

### Effect of community number *K*

Parameter *K* is the number of communities in the NMF method. On varying the value of *K*, different fBN structures/communities are observed. Increasing the number *K* allows us to represent more and more functional communities formed in the brain (refer to Table 2 with *K* = 15). However, as the number of communities increases, we move from underfitting to overfitting community structures, i.e., we face the trade-off between approximating complex brain structures and overfitting them, potentially capturing noise or redundant brain networks.

By starting with a large *K* (say 15, which is possibly double the number of communities in human brain), the effect of considering a higher number of communities can be accounted by ignoring redundant number of communities that correspond to similar fBNs. For example, four communities in Table 2 are identical and correspond to default mode and subcortical networks.

## Discussion

In this paper, we propose LR-MVRC method to build functional connectivity using sparsity and low rank constraints. It is a multivariate FC estimation method that has attracted great attention in the brain fMRI literature recently [8–10]. As a matter of fact, CORR method which exists for FC estimation for almost two decades, considers a pair of two regions while studying functional connection between them. However, this pairwise relationships only reflects the second-order relationships between brain regions without accounting for high-order relationships which is crucial for understanding complex brain networks architecture. To this end, the proposed multivariate method considers all regions simultaneously and regresses one region’s time series onto all other regions’ time series and hence, is named as multivariate regression framework. In addition, besides identifying non-overlapping fBNs, we have used NMF in this work to identify overlapping functional community structure using the resting-state fMRI data.

Experimental results show that the proposed framework is capable of identifying underlying overlapping fBN organization of the human brain. We observe that the proposed method results in multiple fBNs such as Visual Network (VN), Somato-motor Network (SMN), Auditory Network (AN), Cognitive Control Network (CCN), Motor Network (MN), Subcortical Network (SCN), and Default Mode Network (DMN). These networks in resting state fMRI have been consistently identified in previous studies ([46, 47] and references within).

In this paper, we adopted region parcellation and numbering corresponding to the 90 AAL ROIs for the definition of nodes and subsequent construction of fBNs. Multiple studies in fMRI have utilized different node definitions by using clustering or ICA-based methods. The nodes in these studies mainly include the default mode, cognitive control, visual, auditory, subcortical and bilateral limbic networks, which are largely in accordance with the communities identified by LR-MVRC method in this paper.

More importantly, the overlapping communities derived by LR-MVRC with NMF are neuro-physiologically meaningful and comparable to the communities derived using the other most famous modularity optimization methods in the fMRI literature. On visualizing results, we observe that most communities detected by NMF on LR-MVRC FC matrix also appear in the results derived by modularity optimization. However, the most prominent difference is that NMF is able to capture more realistic communities because it allows overlapping communities, say, in default mode, subcortical, bilateral limbic and visual networks. In particular, the identified overlapping regions are mostly found to be the part of frontal, parietal, and temporal brain regions. Similar findings have been previously reported in a few recent studies [48, 49]. On the other hand, the sensory-motor and auditory networks are observed to be formed as disjoint communities as regions associated with these networks are being reported to have high within connectivity [50–52].

ICA method works directly on fMRI time series and produces independent components or activation maps. ICA based analysis requires expertise for rejecting components corrupted with ventricle effects, movement artifacts and sparsely-distributed noises. For example, if *K* or the number of ICA components is large, it requires to discard extra noisy components (components that may not be relevant to contain fBNs). In general, the findings can be contested, particularly, if these are to be used in clinical applications because one may reject wrong components. In this context, LR-MVRC with NMF provides a complementary method to investigate overlapping community structure of the human brain without going through such a dilemma.

### Limitation and future work

The overlapping community structure explored by the proposed method can result in a better understanding of the group-level functional brain networks. However, capturing and analysis of individual subject’s differences could be more useful in understanding individual brain architecture.

A limitation of NMF method is that the performance of NMF depends on its initialization of *K* to some extent. In this paper, the value of *K* in NMF algorithm is initialized based on our prior knowledge of the number of fBNs. Although this is the most popular and simple initialization strategy for NMF methods, it is still a constraint of this algorithm compared to the conventional community detection methods, such as modularity optimization, that do not require any pre-specified *K*. These alternative strategies may lead to faster convergence of the algorithm. Thus, there should be an attempt to auto-detect the number of communities *K* in NMF algorithm.

In this work, thresholded membership value of nodes is used as a deciding factor for the consideration of nodes in the corresponding community. However, this approach does not provide a clear node membership to each overlapping community. Therefore, methods accounting for crisp partitions, allowing each nodes’ contribution to multiple communities are required to be considered in the future works. A few studies have attempted to consider this [53, 54], although these methods do not provide information about nodes’ membership to each community. Owing to this, more sophisticated methods that combine the advantages of both the above mentioned approaches can provide more useful information about the fBNs architecture [24].

Finally, the functional brain imaging data alone may not be sufficient to gain a comprehensive understanding of the brain’s functional organization. A multimodal data combination and analysis of human brain may provide better knowledge about the underlying networks’ organization and guide us to a deeper understanding of the human brain.

## Conclusions

In this work, we propose a Low Rank Multivariate Vector Regression-based Connectivity (LR-MVRC) method for estimating FC matrix. Proposed method utilizes both sparsity and low rank constraints while estimating the FC matrix. Most of the previous studies extract disjoint communities or functional brain networks, we extract overlapping communities via NMF that may be biologically more correct for understanding human brain’s functional organization. Experimental results suggest that the proposed framework can better characterize the brain networks’ organization at the group level. In conclusion, we believe that the proposed method and its potential applications could provide new insights into the functional networks’ organization of the human brain.
